# Case of Fracture of the Spine. Account of the Post-Mortem Examination of the Body of Patrick Connell, upon Whom Mr. Brodie Performed the Operation of Tying the External Iliac Artery, in the Year 1828

**Published:** 1831-10

**Authors:** Jas. Laidlaw

**Affiliations:** Surgeon.


### FRACTURE OF THE SPINE.

*Case of Fracture of the Spine. Account of the Post-mortem Examination of the Body of Patrick Connell, upon whom* Mr. BRODIE *performed the Operation of Tying the External Iliac Artery, in the year* 1828. By JAS. LAIDLAW, Esq. Surgeon.

—— WRIGHT, upwards of sixty years of age, by trade a stonemason, some years ago received a severe injury of the loins while at work: from this he recovered after some time, so as to be able again to follow his laborious occupation.

In the early part of the present year, he received another accidental injury from a severe blow on the sternum, and continued ill for above a month, when he died apparently from effusion in the chest. Upon examining the body, it was found that the cartilage of the first rib had become ossified, and, having been fractured by the injury, its broken, ragged ends had produced so much irritation, that a large abscess had formed in the superior lobe of the left lung, and which had been the immediate cause of his death.

Being aware of the injury to the loins which he had received formerly, I determined upon examining the spine with attention, and for that purpose removed a considerable portion of it, with the sacrum attached. Upon removing the soft parts, it was found that the spinous processes of the second, third, and fourth lumbar vertebræ had been broken off: the spinous process of the second had never united, but had formed a joint at the broken part, so as to be moved easily upward and downward; the spinous processes of the third and fourth had both attached themselves to the fourth vertebræ, and were firmly united to it by bone, so that the third vertebra, when separated from the others, appears to be left entirely without a spinous process. The spinal canal, instead of being, as usual in the loins, somewhat larger than in the back, was in this case a little smaller.

The injury appeared not to have impaired, in the least, any of the powers of the patient, as he continued, up to the period of his last illness, to work very industriously at his trade. It would, of course, have been desirable to state the symptoms, or rather the effect produced at the time by the injury to the loins; but the above is the only information I could obtain of the case.

Patrick Connell was a patient under the care of Mr. Brodie, in St. George's Hospital, for inguinal aneurism, in the early part of the year 1828, and for which it was found necessary to tie the external iliac artery, which was accordingly performed with success by Mr. Brodie, on the 21st of February in that year. As the details of this case have been published in this Journal, (April 1828, p. 328,) I presume that the following account of the state of the patient subsequent to the operation, and of the appearances observed on dissection, will be interesting to your readers.

Since the performance of the operation, the patient, who had always been very unhealthy, and had been exposed to many hardships, has never been completely well, constantly suffering from cough and affection of the lungs; his legs became œdematous; and continuing thus to get worse, in the early part of the present year he became dropsical and died.

Upon examining the remains of the aneurismal tumor, it was found to be somewhat larger than a pigeon's egg, situated immediately below Poupart's ligament, and filled with a firm coagulum. Upon tracing the vessels connected with it, it was found that in this case the external iliac artery, instead of, as usual, giving off the epigastric and circumflexa ilii, and then becoming the common femoral artery, divided all at once into three large vessels; one of which again dividing, formed the epigastric and circumflexa ilii; another formed the profunda femoris, and the third continued its course as the superficial femoral artery. It was at this point of general division that the aneurism had formed, so that, in the preparation, the cut ends of several vessels are seen hanging from the tumor. The external iliac artery was entirely obliterated, from about three-quarters of an inch above the tumor; the internal iliac was nearly twice as large as it is in ordinary circumstances, but appeared to be perfectly healthy; nor was there any appearance of disease found in the aorta. The operation had been most completely successful, and it would have been satisfactory to trace the anastomosis of the vessels; but the relatives of the deceased having a great objection to allowing an examination, it was necessary to perform it clandestinely, and the incision having been made in the loins only sufficiently large to admit the hand, and so remove the preparation, any further dissection was impossible.

